# Neoadjuvant chemoradiotherapy with or without PD-1 inhibitors in MMR−proficient non−metastatic rectal cancer: a meta-analysis of randomized controlled trials

**DOI:** 10.3389/fimmu.2026.1792283

**Published:** 2026-03-03

**Authors:** Yuegang Li, Chengcheng Han, Jianqiang Tang

**Affiliations:** 1 Department of Surgical Oncology, First Affiliated Hospital of China Medical University, Shenyang, China; 2 Department of Endoscopy, National Cancer Center/National Clinical Research Center for Cancer/Cancer Hospital, Chinese Academy of Medical Sciences and Peking Union Medical College, Beijing, China; 3 Department of Colorectal Surgery, National Cancer Center/National Clinical Research Center for Cancer/Cancer Hospital, Chinese Academy of Medical Sciences and Peking Union Medical College, Beijing, China

**Keywords:** immunotherapy, meta-analysis, neoadjuvant therapy, pMMR/MSS, rectal cancer

## Abstract

**Aim:**

In proficient mismatch repair (pMMR) non metastatic rectal cancer, standard neoadjuvant chemoradiotherapy (nCRT) yields low pathological and clinical complete response rates. Early randomized trials suggest adding PD 1 inhibitors may increase response but randomized evidence has not been synthesized.

**Methods:**

We performed a systematic review and meta-analysis of phase II–III randomized trials comparing nCRT plus PD 1 inhibitor versus nCRT alone in adults with untreated pMMR non metastatic rectal cancer. PubMed, Web of Science, Embase and CENTRAL were searched to 30 Sept 2025. Two reviewers extracted data. Dichotomous outcomes were pooled as risk ratios (RRs) with 95% confidence intervals (CIs) using a DerSimonian–Laird random effects model; heterogeneity was assessed by I2. Prespecified subgroup analyses compared short course versus long course radiotherapy.

**Results:**

Six trials (n=935; nCRT+PD 1 = 461; nCRT=474) were included; agents evaluated included pembrolizumab, sintilimab, tislelizumab and camrelizumab. PD 1 addition significantly increased pathological complete response (pCR) (RR 1.79, 95% CI 1.34–2.40) and showed a non-definitive increase in clinical complete response (cCR) (RR 1.67, 95% CI 0.89–3.13). No clear differences were seen for R0 resection, sphincter preservation, grade ≥3 neoadjuvant toxicity, or surgery related adverse events. Subgroup analysis suggested greater pCR benefit with short course radiotherapy.

**Conclusion:**

Among patients with pMMR non−metastatic rectal cancer, adding PD−1 inhibitors to standard nCRT improves pCR—most markedly when combined with short−course radiotherapy—with no statistically significant increase detected in high−grade neoadjuvant toxicity or major surgical morbidity. These randomized data support progression to confirmatory phase III trials to define optimal sequencing, regimen standardization and long−term oncologic and functional outcomes.

**Systematic Review Registration**: https://www.crd.york.ac.uk/prospero/**, identifier 420251137668.**

## 1 Introduction

Rectal cancer remains a common gastrointestinal malignancy, with approximately 30%–40% of patients presenting with locally advanced disease (1). Standard management for locally advanced rectal cancer includes neoadjuvant chemoradiotherapy (nCRT) followed by total mesorectal excision (TME); more recently, total neoadjuvant therapy (TNT) has been adopted to increase pathological complete response (pCR) rates and improve systemic disease control (2, 3). Despite these advances, pCR after nCRT or TNT in mismatch repair–proficient (pMMR) non metastatic rectal cancer generally remains below 20%, and clinical complete response (cCR) rates are typically 15%–30%, leaving substantial unmet need for strategies that increase organ preservation and long-term disease control (4, 5).

Programmed cell death protein 1 (PD−1) inhibitors produce durable antitumor responses in several solid tumors and show marked activity in colorectal cancers with deficient mismatch repair (dMMR) or high microsatellite instability (MSI−H) (6). Those phenotypes, however, represent a minority of colorectal cancers overall and an even smaller fraction of locally advanced rectal cancers, while the majority of tumors are pMMR or microsatellite stable (MSS) and derive limited benefit from PD−1 monotherapy, likely because of low baseline CD8+ T−cell infiltration and an immunologically “cold” tumor microenvironment (7). Preclinical and early clinical data indicate that radiotherapy and certain chemotherapies can increase tumor immunogenicity by promoting antigen release, dendritic cell activation, and T−cell recruitment, providing a biological rationale for combining PD−1 blockade with nCRT to convert non−inflamed tumors into responders (8).

Several early−phase clinical trials have tested PD−1 inhibitors added to standard nCRT in pMMR non−metastatic rectal cancer with promising signals for increased tumor response, but results have been heterogeneous and no pooled quantitative synthesis of randomized data is available (9–11). We therefore performed a systematic review and meta−analysis of phase II and III randomized controlled trials to evaluate whether adding PD−1 inhibitors to standard nCRT improves pCR and cCR rates without substantially increasing high−grade toxicity or surgical morbidity, and to explore whether radiotherapy strategy (short−course versus long−course) modifies the treatment effect.

## 2 Materials and methods

### 2.1 Study design and registration

This systematic review and meta-analysis was conducted and reported in accordance with the Preferred Reporting Items for Systematic Reviews and Meta Analyses (PRISMA) statement. The study protocol was registered prospectively in PROSPERO (CRD 420251137668).

### 2.2 Data sources and search strategy

We searched PubMed Web of Science, Embase, and the Cochrane Central Library from database inception through 30 September 2025 without language restrictions. The full electronic search strategies for each database are provided in **Supplementary Table 1** and include controlled vocabulary (MeSH/Emtree) and free−text terms for “rectal cancer, “ “neoadjuvant chemoradiotherapy, “ “PD−1, “ and randomized trial filters.

### 2.3 Eligibility criteria

We included phase II or phase III randomized controlled trials that enrolled adults (≥18 years) with previously untreated, non-metastatic rectal cancer confirmed as pMMR by local or central testing. Eligible trials randomized participants to (1) nCRT plus a PD 1 inhibitor versus (2) nCRT alone, and reported at least one prespecified outcome: pCR, cCR, R0 resection rate, sphincter preservation rate, neoadjuvant treatment related adverse events, or surgery related adverse events. Trials combining investigational agents beyond PD 1 inhibitors were excluded. For the purposes of this analysis, we used the term nCRT broadly to refer to all neoadjuvant regimens that included both radiotherapy and chemotherapy prior to surgery, including regimens that meet the criteria for total neoadjuvant therapy (TNT).

### 2.4 Study selection, data extraction, and quality assessment

Two reviewers independently screened titles and abstracts, retrieved full texts for potentially eligible records, and resolved disagreements by consensus or third−party arbitration. Using a prespecified standardized form, we extracted trial identifiers, phase, country, randomization method, sample size, treatment regimens (chemotherapy agents, PD−1 agent, radiotherapy dose and fractionation), definitions of pCR and cCR, follow−up duration, and outcome counts for each arm. Risk of bias was independently assessed by two reviewers with the Cochrane RoB 2 tool across five domains (randomization process; deviations from intended interventions; missing outcome data; outcome measurement; selective reporting), with discrepancies adjudicated by a third reviewer. The risk−of−bias traffic−light plot is provided in **Supplementary Figure 1**. For safety outcomes, we extracted the number of patients experiencing Grade 1–2 and Grade ≥3 adverse events during neoadjuvant treatment and within 30 days after surgery, as reported in each trial. These were categorized as treatment-related or surgery-related adverse events according to the original study definitions. Pooled risk ratios (RRs) were calculated for each AE category using a random-effects model.

## 3 Statistical analysis

All analyses were performed in R (version 4.4.1) using the readxl, dplyr, tidyr, meta, and grid packages. Outcome data were imported from an Excel workbook and reshaped to wide format with pivot_wider (tidyr). Dichotomous endpoints (pCR, cCR, R0 resection, sphincter preservation, neoadjuvant treatment-related adverse events, or surgery-related adverse events) were pooled as RRs with 95% confidence intervals (CIs) using the metabin function (meta package) under an inverse-variance–weighted DerSimonian–Laird random-effects model. For outcomes with rare or zero events, a continuity correction of 0.5 was applied where appropriate to enable RR estimation. Sensitivity analyses were additionally performed using the Hartung–Knapp adjustment and REML estimator to assess robustness in the context of small-study meta-analysis. Between-study heterogeneity was quantified by the I² statistic. Forest plots, including 95% CIs and prediction intervals, were generated via the forest function and exported to PDF. Subgroup analyses were prespecified for radiotherapy modality (short course versus long course radiotherapy). All tests were two-sided, with p < 0.05 denoting statistical significance.

## 4 Results

### 4.1 Search results and study characteristics

Six randomized controlled trials (12–17) (n = 935) met inclusion criteria (study selection flowchart, **Supplementary Figure 2**): nCRT + PD-1, n = 461; nCRT alone, n = 474. Three trials reported patients managed with a watch-and-wait strategy after achieving cCR (nCRT + PD-1, n = 39; nCRT alone, n = 23). Investigated PD-1 agents were pembrolizumab (1 trial), sintilimab (3 trials), tislelizumab (1 trial), and camrelizumab (1 trial). Radiotherapy regimens comprised long-course CRT (50.4 Gy in 28 fractions; 3 trials) and short-course RT (25 Gy in 5 fractions; 3 trials). Key trial designs, baseline characteristics, and outcome definitions are summarized in the **Table 1**.

**Table 1:** Characteristics of randomized clinical trials and outcomes included in the meta-analysis.

Source	Nation	Phase	ClinicalTrials.gov Identifier	Stage	No. of pMMR	Intervention vs. control	cCR, n/N	Surgical Cases, n/N	pCR, n/N	R0 Resection, n/N	Sphincter-preserving Surgery, n/N
NRG-GI002 (2021)	USA	II	NCT02921256	cT1–4 N0/+	90	FOLFOX×6**→**LCRT→Pembro×6→TME	11/79	69	22/69	65/69	41/69
					95	FOLFOX×6**→**LCRT→TME	11/81	68	20/68	61/68	49/68
Xu et al. (2024)	China	II	NCT04304209	cT2–4 N0/+	67	(CAPOX + Sintili)×4→LCRT→TME	9/66	57	21/57	–	54/57
					67	CAPOX×4→LCRT→TME	2/65	56	16/56	–	51/56
POLARSTAR (2025)	China	II	NCT05245474	cT1–4 N0/+	48	LCRT→Tislelizu×3→TME	–	48	15/48	46/48	44/48
					49	LCRT→TME	–	49	6/49	37/49	33/49
UNION (2024)	China	III	NCT04928807	cT2–4 N0/+	105	SCRT→(Camre + CAPOX)×2→TME	–	105	42/105	–	–
					112	LCRT→CAPOX×2→TME	–	112	18/112	–	–
SRELLAR II (2025)	China	II/III	NCT05484024	cT2–4 N+	110	SCRT→(Sintili + CAPOX)×4→TME	19/110	72	31/72	70/72	55/72
					108	SCRT→CAPOX×4→TME	10/108	82	17/82	80/82	63/82
SPRING-01 (2025)	China	II	ChiCTR2100052288	cT2–4 N0/+	41	SCRT→(Sintili + CAPOX)×6→TME	–	41	24/41	–	–
					43	SCRT→CAPOX×6 →TME	–	43	12/43	–	–

TME, Total Mesorectal Excision; SCRT, short-course radiotherapy; LCRT, Long-course chemoradiotherapy; cCR, clinical complete response; pCR, pathological complete response; Pembro, Pembrolizumab; Sintili, Sintilimab; Tislelizu, Tislelizumab; Camre, Camrelizumab; Arrows indicate treatment sequence. LCRT was generally delivered as 45–50.4 Gy in 25–28 fractions with concurrent capecitabine, while SCRT was delivered as 25 Gy in 5 fractions followed by systemic therapy. All pCR definitions were based on ypT0N0 unless otherwise specified.

### 4.2 Efficacy

**Figure 1:**
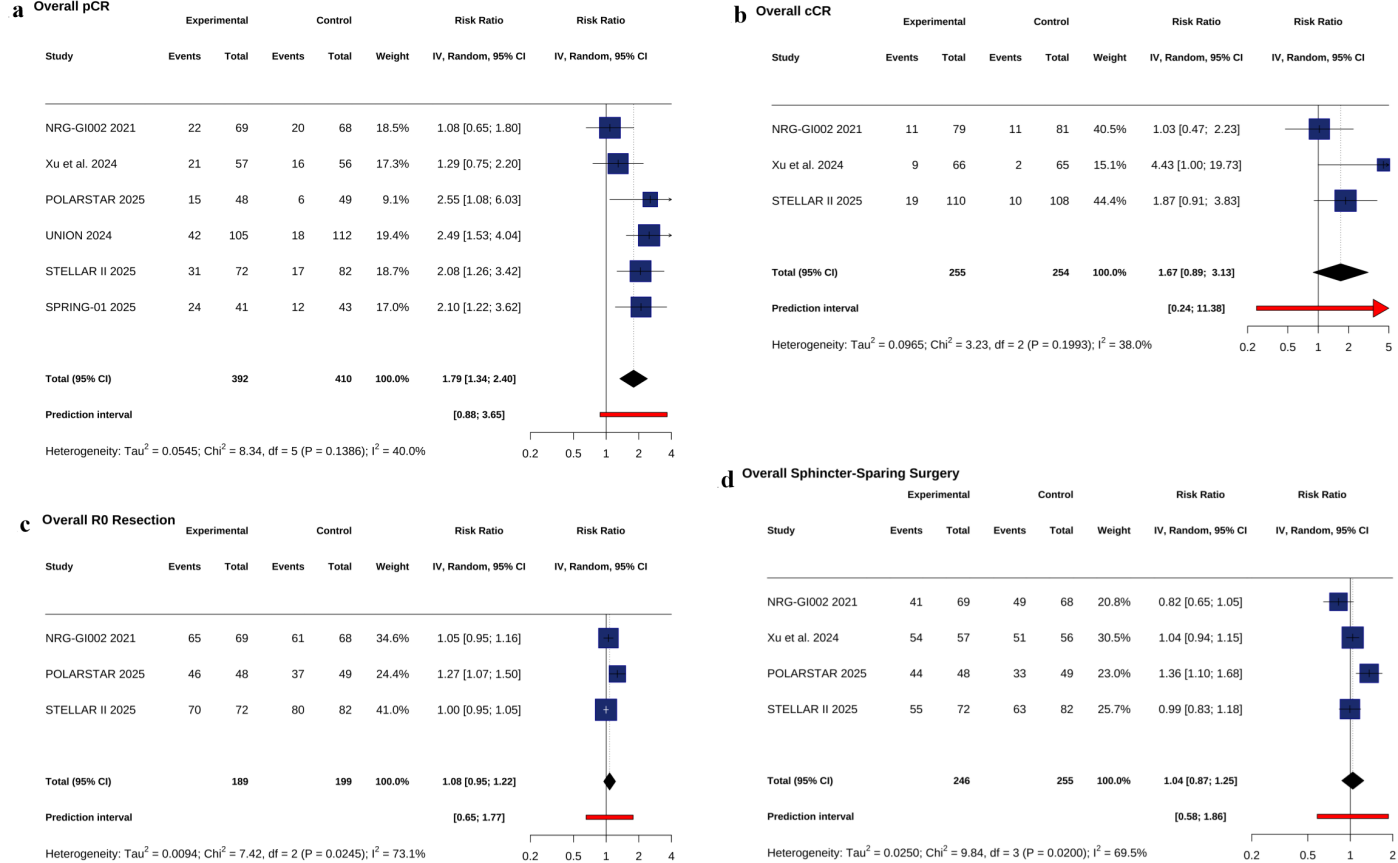
Forest plot of pCR rate **(a)**, Forest plot of cCR rate **(b)**, Forest plot of R0 resection rate **(c)**, Forest plot of sphincter-preserving surgery **(d)**.

**Figure 2:**
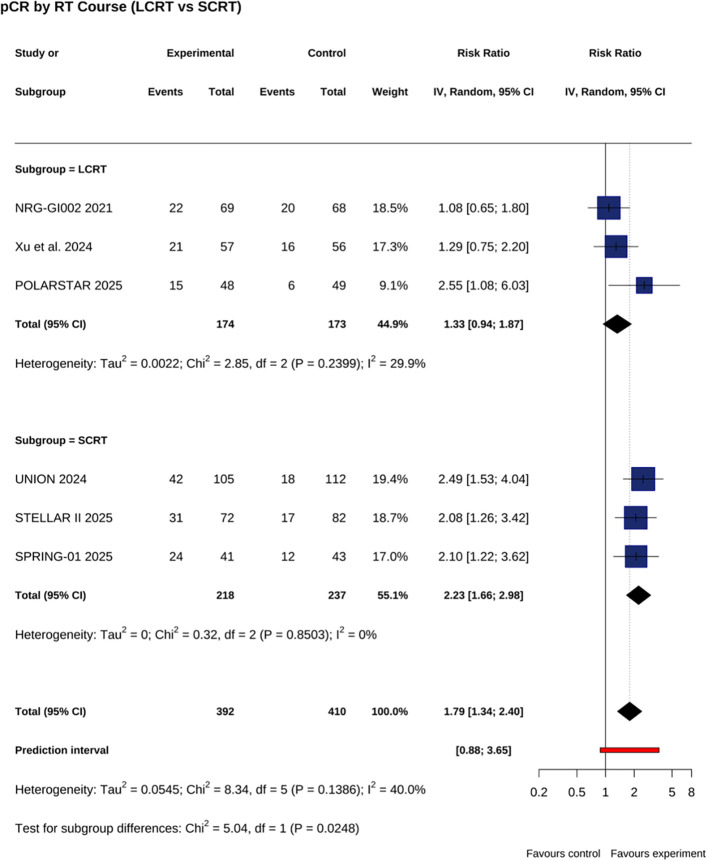
Subgroup analysis based on radiotherapy regimens: pCR comparison of SCRT versus LCRT.

Addition of PD 1 inhibitors to nCRT significantly increased pCR rate (RR = 1.79; 95% CI, 1.34–2.40; P <.001; I² = 40%) (**Figure 1a**). A non-significant trend was observed for cCR (RR = 1.67; 95% CI, 0.89–3.13; P = .11; I² = 38%) (**Figure 1b**). No significant differences were seen for R0 resection (RR = 1.08; 95% CI, 0.95–1.22; P = .23; I² = 73.1%) or sphincter preservation (RR = 1.04; 95% CI, 0.87–1.25; P = .67; I² = 69.5%) (**Figures 1c, d**). In prespecified radiotherapy strategy subgroup analyses, the pCR benefit was greater with short course RT (RR = 2.23; 95% CI, 1.66–2.98; P <.001; I² = 0%) than with long course CRT (RR = 1.33; 95% CI, 0.94–1.87; P = .12; I² = 29.9%); test for interaction: χ² = 5.04; df = 1; P = .02 (**Figure 2**). This finding remained robust in sensitivity analysis using the Hartung–Knapp adjustment and REML estimator (RR = 0.58; 95% CI, 0.21–0.96; P = .010), as shown in **Supplementary Table 2**.

### 4.3 Safety

**Figure 3:**
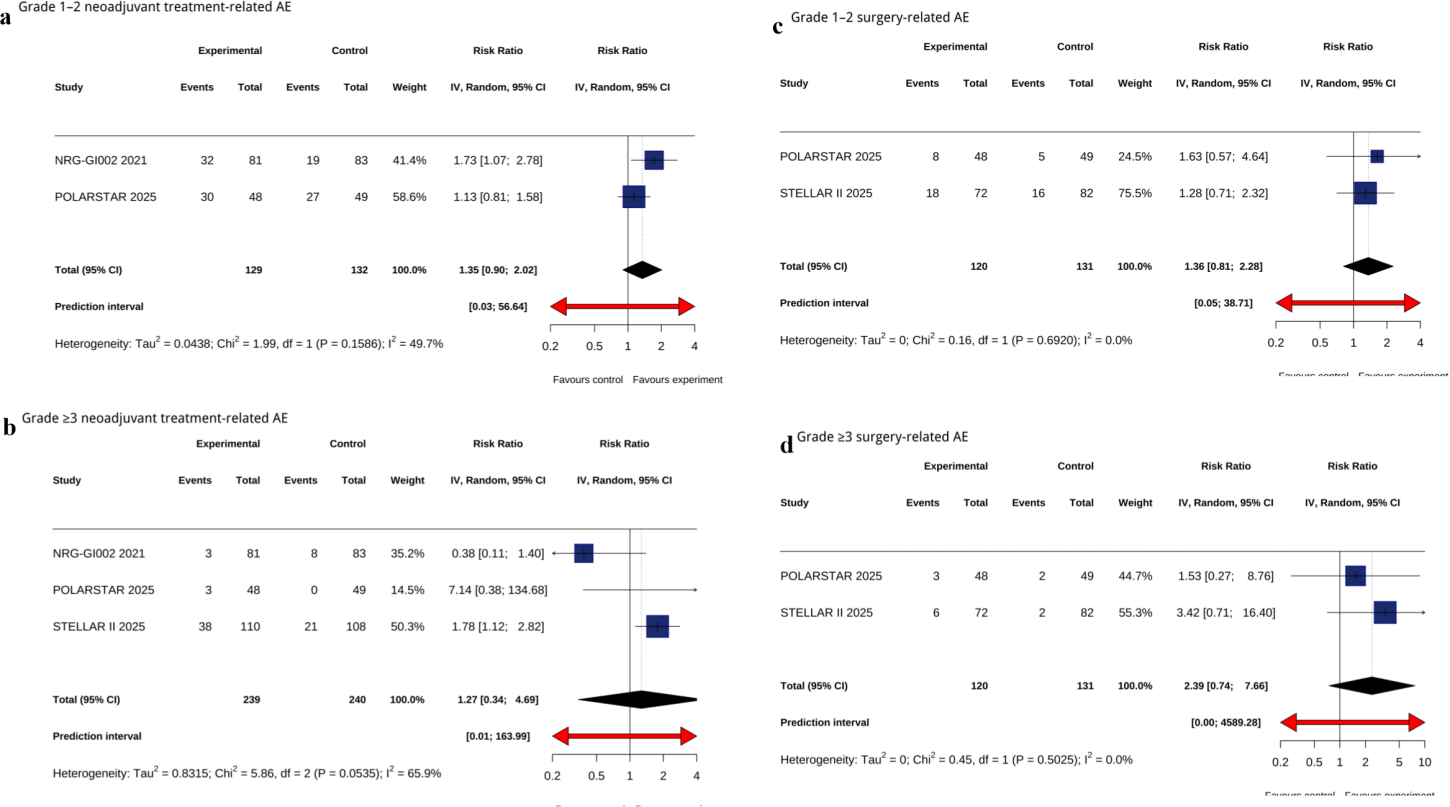
Forest plot of Grade 1–2 neoadjuvant treatment-related AE **(a)**, Forest plot of Grade ≥3 neoadjuvant treatment-related AE **(b)**, Forest plot of Grade 1–2 surgery-treated AE **(c)**, Forest plot of Grade ≥3 surgery-treated AE **(d)**.

The combination arm showed a numerically higher risk of Grade 1–2 neoadjuvant treatment-related adverse events (RR = 1.35; 95% CI, 0.90–2.02; P = .15; I² = 49.7%), but this difference was not statistically significant(**Figure 3a**). No statistically significant difference was detected for Grade ≥3 neoadjuvant treatment-related adverse events (RR = 1.27; 95% CI, 0.34–4.69; P = .72; I² = 65.9%) (**Figure 3b**). For surgical morbidity, non-significant differences were observed in Grade 1–2 surgery-related adverse events (RR = 1.36; 95% CI, 0.81–2.28; P = .25; I² = 0%) and no significant elevation in Grade ≥3 surgery-related adverse events (RR = 2.39; 95% CI, 0.74–7.66; P = .15; I² = 0%) (**Figures 3c, d**). However, the wide confidence intervals and limited number of events suggest that these safety findings should be interpreted with caution.

## 5 Discussion

This meta-analysis pooled data from six randomized controlled trials and, for the first time in a quantitative synthesis, demonstrated that adding PD-1 inhibitors to nCRT significantly increases pCR rates in pMMR non metastatic rectal cancer. A greater pCR benefit was observed in the short-course radiotherapy subgroup; however, given the limited number of trials, this finding should be considered hypothesis-generating and warrants further investigation in future prospective studies. The pCR improvement was not accompanied by a significant increase in grade ≥3 toxicity or surgical complications, suggesting that the combined approach has an acceptable tolerability and safety profile. However, adverse event reporting was highly heterogeneous across studies, with differences in grading criteria, attribution, and reporting timepoints. Many trials did not distinguish between immune-related and chemoradiotherapy-related toxicities, and some reported adverse events across the entire treatment course without separating neoadjuvant and adjuvant phases. Due to these inconsistencies, we did not perform pooled analyses of specific adverse event types. This limitation highlights the need for future trials to adopt standardized definitions and timepoint-specific AE reporting to enable more meaningful safety comparisons.

Radiotherapy can induce immunogenic cell death within tumors, releasing tumor antigens and promoting dendritic cell activation, thereby increasing the pool of effector T cell targets for PD-1 blockade. Short-course, high fraction radiotherapy may provoke a stronger local immune activation and a more intense inflammatory response in the tumor microenvironment, which could explain the greater pCR improvement compared with long-course CRT (18).

Clinically, pCR is an important predictor of postoperative survival and organ preservation. The observed increase in pCR in our analysis may expand eligibility for watch and wait strategies and sphincter preserving procedures, although the impact on long-term oncologic and functional outcomes remains to be established. Interestingly, while the addition of PD-1 inhibitors significantly improved pCR rates, the increase in cCR did not reach statistical significance. This discrepancy may reflect differences in the sensitivity and specificity of clinical versus pathological response assessments. Clinical complete response is typically determined by a combination of endoscopy, digital rectal examination, and imaging, which may underestimate residual microscopic disease or be influenced by interobserver variability. In contrast, pCR is based on histopathologic examination of the resected specimen and provides a more definitive assessment of tumor eradication. These findings underscore the need for standardized and validated criteria for cCR assessment, particularly in the context of organ-preserving strategies such as watch-and-wait. Future studies should explore whether improved pCR with immunoradiotherapy translates into more reliable cCR detection and durable non-operative management.

Although the included trials used different PD-1 agents and radiotherapy fractionation schemes, the consistent efficacy signal supports the generalizability of a radiotherapy–immunotherapy synergistic effect. Radiotherapy dose, fractionation, and sequencing with chemotherapy or PD-1 inhibitors varied across studies. LCRT was generally delivered as 45–50.4 Gy in 25–28 fractions with concurrent capecitabine, while SCRT was delivered as 25 Gy in 5 fractions followed by systemic therapy. These differences may influence the degree of immune activation and contribute to clinical heterogeneity. Moreover, several included regimens incorporated multi-cycle chemotherapy before or after radiotherapy and may be considered TNT, which could further contribute to clinical heterogeneity. Given the small number of included trials, further stratified analyses by chemotherapy regimen, PD-1 agent, or sequencing were not feasible. However, these factors likely contribute to the observed heterogeneity and should be addressed in future trials with harmonized protocols. Future work should integrate biomarkers such as tumor mutational burden, PD L1 expression, and measures of immune infiltration to more precisely identify pMMR patients most likely to benefit from combination therapy (19).

Further studies are needed to define optimal sequencing and dosing—whether concurrent with radiotherapy, as post radiotherapy maintenance, or as consolidation after chemoradiotherapy—and should prioritize long term endpoints including disease free survival, overall survival, and functional organ preservation. Multi omics and single cell sequencing approaches will be valuable for elucidating the dynamic effects of combined radiotherapy and immunotherapy on the tumor microenvironment (20).

The main strength of this analysis is its adherence to PRISMA guidelines and the Cochrane assessment framework, systematically synthesizing available multicenter randomized trial data. However, the predominance of early-phase (phase II) trials with limited sample sizes and exploratory designs introduces phase-related heterogeneity, which may affect the maturity and generalizability of the findings. Moreover, most studies lacked long-term oncologic endpoints such as disease-free and overall survival, as well as functional outcomes like sphincter preservation, limiting the ability to assess the durability and clinical relevance of the observed pCR improvements. These limitations underscore the need for confirmatory phase III trials with standardized treatment protocols, longer follow-up, and comprehensive outcome reporting to validate the benefits of immunoradiotherapy in pMMR rectal cancer.

## 6 Conclusion

Adding PD-1 inhibitors to nCRT appears to safely increase pCR rates in pMMR non metastatic rectal cancer, with the benefit most pronounced in the short course radiotherapy setting. These results provide a rationale for designing and conducting phase III trials and support the further clinical investigation of immunoradiotherapy combinations in the neoadjuvant treatment of rectal cancer, with particular emphasis on long-term survival, functional outcomes, and quality of life.

## Data Availability

The original contributions presented in the study are included in the article/**Supplementary Material**. Further inquiries can be directed to the corresponding author.

## Supplementary material

Supplementary Figure 1 Flow diagram of study selection and literature search results.

Supplementary Figure 2 Randomized Clinical Trial Risk of Bias Assessment.
