# Supplementary figures and images for: Neoadjuvant chemoradiotherapy with or without PD-1 inhibitors in MMR−proficient non−metastatic rectal cancer: a meta-analysis of randomized controlled trials
Source: Front Immunol. 2026 Mar 3;17:1792283. doi: 10.3389/fimmu.2026.1792283 (PMC12992012; doi:10.3389/fimmu.2026.1792283)

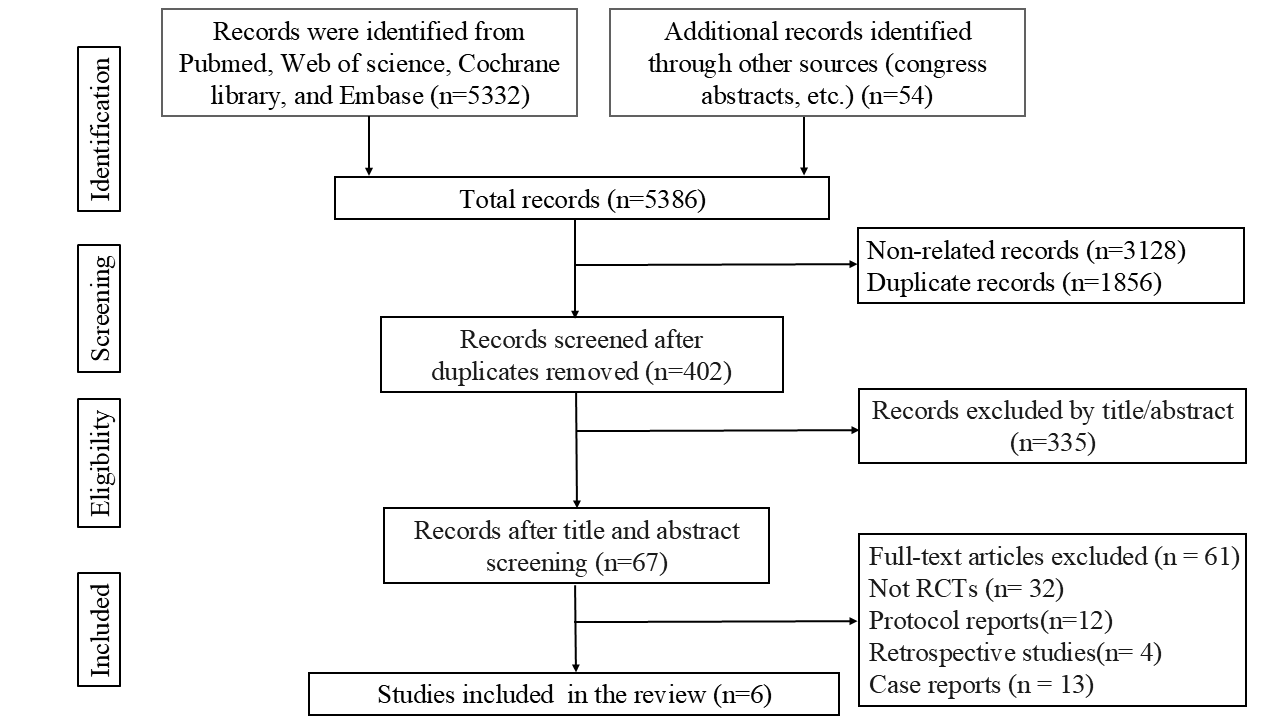

Supplement: Supplementary Figure 1 — Flow diagram of study selection and literature search results. [file Image1.tif]

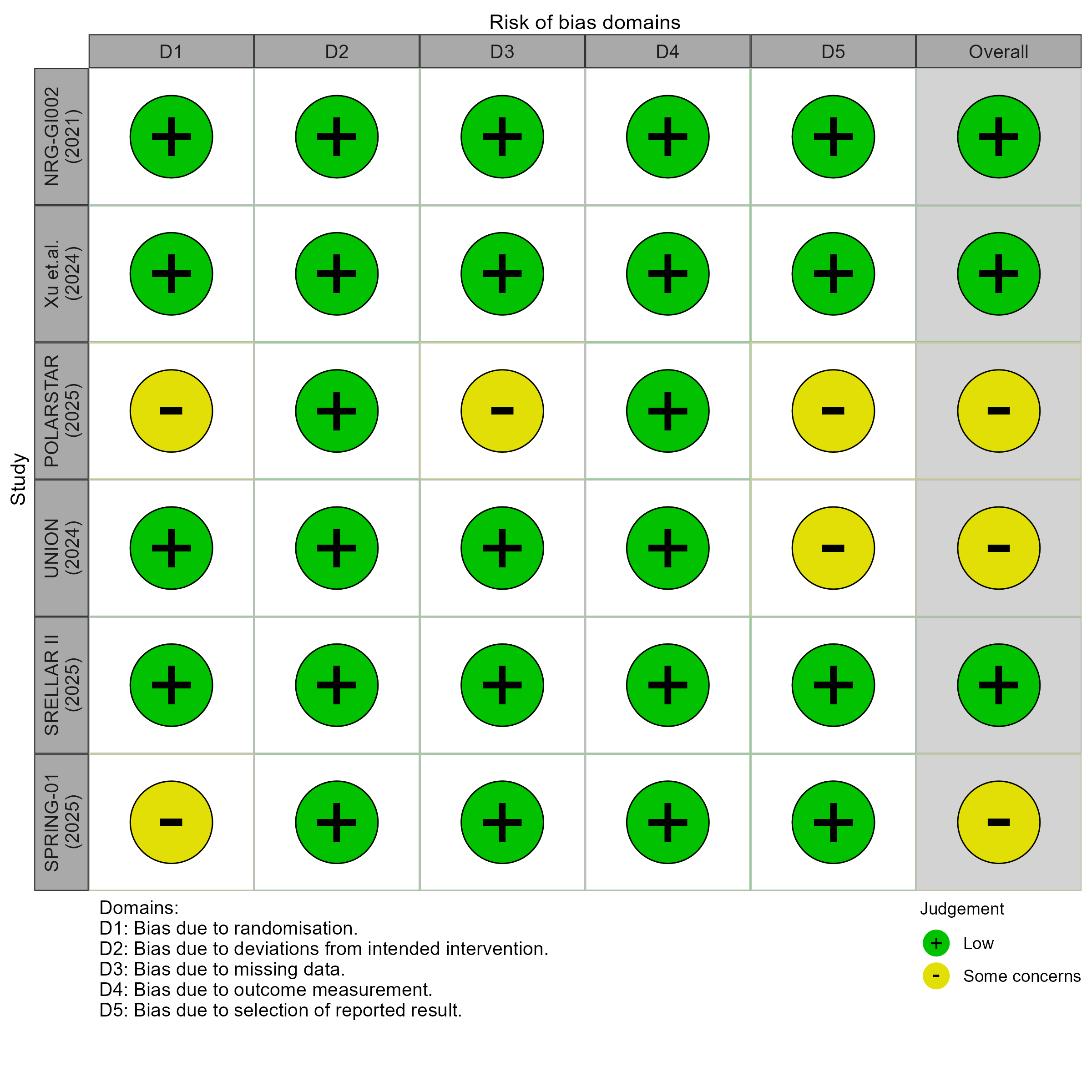

Supplement: Supplementary Figure 2 — Randomized Clinical Trial Risk of Bias Assessment. [file Image2.tiff]
