# Supplementary material for: Neoadjuvant chemoradiotherapy with or without PD-1 inhibitors in MMR−proficient non−metastatic rectal cancer: a meta-analysis of randomized controlled trials
Source: Front Immunol. 2026 Mar 3;17:1792283. doi: 10.3389/fimmu.2026.1792283 (PMC12992012; doi:10.3389/fimmu.2026.1792283)
Supplement: Supplementary file 3 [file Table1.docx]

| ***Pubmed*** | ***Search Strategy:*** |  |
| --- | --- | --- |
| #1 | (Rectal Neoplasms[MeSH Terms]) OR (((((((((((((((((Neoplasm, Rectal[Title/Abstract]) OR (Rectal Neoplasm[Title/Abstract])) OR (Neoplasms, Rectal[Title/Abstract])) OR (Rectum Neoplasms[Title/Abstract])) OR (Neoplasm, Rectum[Title/Abstract])) OR (Rectum Neoplasm[Title/Abstract])) OR (Rectal Tumors[Title/Abstract])) OR (Rectal Tumor[Title/Abstract])) OR (Tumor, Rectal[Title/Abstract])) OR (Cancer of Rectum[Title/Abstract])) OR (Rectum Cancers[Title/Abstract])) OR (Cancer of the Rectum[Title/Abstract])) OR (Rectal Cancer[Title/Abstract])) OR (Cancer, Rectal[Title/Abstract])) OR (Rectal Cancers[Title/Abstract])) OR (Rectum Cancer[Title/Abstract])) OR (Cancer, Rectum[Title/Abstract])) | 68782 |
| #2 | (Immunotherapy[MeSH Terms]) OR (((((((((((((((((((((((((((((((((((((((((((((((((((((immunotherapy[Title/Abstract]) OR (immunotherapies[Title/Abstract])) OR (immunotherapeutic[Title/Abstract])) OR (immunotherapeutic agents[Title/Abstract])) OR (checkpoint inhibitor[Title/Abstract])) OR (checkpoint inhibitors[Title/Abstract])) OR (immune checkpoint inhibitors[Title/Abstract])) OR (checkpoint blockade[Title/Abstract])) OR (checkpoint blockades[Title/Abstract])) OR (programmed cell death protein 1[Title/Abstract])) OR (programmed cell death 1[Title/Abstract])) OR (programmed death 1[Title/Abstract])) OR (programmed death protein 1[Title/Abstract])) OR (programmed cell death receptor 1 inhibitor[Title/Abstract])) OR (programmed cell death receptor 1 inhibitor[Title/Abstract])) OR (programmed cell death receptor 1 inhibitors[Title/Abstract])) OR (programmed cell death 1 receptor inhibitor[Title/Abstract])) OR (programmed cell death 1 receptor inhibitors[Title/Abstract])) OR (anti pd 1 antibody[Title/Abstract])) OR (anti pd 1 antibodies[Title/Abstract])) OR (programmed cell death 1 ligand 1[Title/Abstract])) OR (programmed death 1 ligand 1[Title/Abstract])) OR (programmed death ligand 1[Title/Abstract])) OR (PD-1[Title/Abstract])) OR (PD1[Title/Abstract])) OR (PD-L1[Title/Abstract])) OR (PDL1[Title/Abstract])) OR (anti pd l1 antibody[Title/Abstract])) OR (anti pd l1 antibodies[Title/Abstract])) OR (anti pd l1 inhibitor[Title/Abstract])) OR (anti pd l1 inhibitors[Title/Abstract])) OR (pd 1 inhibitors[Title/Abstract])) OR (pd l1 inhibitors[Title/Abstract])) OR (ctla 4 inhibitors[Title/Abstract])) OR (lag 3 inhibitors[Title/Abstract])) OR (tim 3 inhibitors[Title/Abstract])) OR (Nivolumab[Title/Abstract])) OR (Pembrolizumab[Title/Abstract])) OR (Cemiplimab[Title/Abstract])) OR (Tislelizumab[Title/Abstract])) OR (Dostarlimab[Title/Abstract])) OR (Toripalimab[Title/Abstract])) OR (Retifanlimab[Title/Abstract])) OR (Atezolizumab[Title/Abstract])) OR (Avelumab[Title/Abstract])) OR (Durvalumab[Title/Abstract])) OR (Ipilimumab[Title/Abstract])) OR (Tremelimumab[Title/Abstract])) OR (Camrelizumab[Title/Abstract])) OR (Sintilimab[Title/Abstract])) OR (Zimberelimab[Title/Abstract])) OR (Dostarlimab[Title/Abstract])) OR (Spartalizumab[Title/Abstract])) | 516434 |
| #3 | #1 And #2 | 1133 |

| ***Web of science*** | ***Search Strategy:*** |  |
| --- | --- | --- |
| #1 | (((((((((((((((((TS=( Rectal Neoplasms)) OR TS=(Neoplasm, Rectal)) OR TS=(Rectal Neoplasm)) OR TS=(Neoplasms, Rectal)) OR TS=(Rectum Neoplasms)) OR TS=(Neoplasm, Rectum)) OR TS=(Rectum Neoplasm)) OR TS=(Rectal Tumors)) OR TS=(Rectal Tumor)) OR TS=(Tumor, Rectal)) OR TS=(Cancer of Rectum)) OR TS=(Rectum Cancers)) OR TS=(Cancer of the Rectum)) OR TS=(Rectal Cancer)) OR TS=(Cancer, Rectal)) OR TS=(Rectal Cancers)) OR TS=(Rectum Cancer)) OR TS=(Cancer, Rectum) | 156051 |
| #2 | ((((((((((((((((((((((((((((((((((((((((((((((((((TS=(Immunotherapy)) OR TS=(immunotherapies)) OR TS=(immunotherapeutic)) OR TS=(immunotherapeutic agents)) OR TS=(checkpoint inhibitor)) OR TS=(checkpoint inhibitors)) OR TS=(immune checkpoint inhibitors)) OR TS=(checkpoint blockade)) OR TS=(checkpoint blockades)) OR TS=(programmed cell death protein 1)) OR TS=(programmed cell death 1)) OR TS=(programmed death 1)) OR TS=(programmed death protein 1)) OR TS=(programmed cell death receptor 1 inhibitor)) OR TS=(programmed cell death receptor 1 inhibitor)) OR TS=(programmed cell death receptor 1 inhibitors)) OR TS=((programmed cell death 1 receptor inhibitor)) OR TS=(anti pd 1 antibody)) OR TS=(anti pd 1 antibodies)) OR TS=(programmed cell death 1 ligand 1)) OR TS=(programmed death 1 ligand 1)) OR TS=(programmed death ligand 1)) OR TS=(PD-1)) OR TS=(PD1)) OR TS=(PD-L1)) OR TS=(PDL1)) OR TS=(anti pd l1 antibody)) OR TS=(anti pd l1 antibodies)) OR TS=(anti pd l1 inhibitor)) OR TS=(anti pd l1 inhibitors)) OR TS=(pd 1 inhibitors)) OR TS=(pd l1 inhibitors)) OR TS=(ctla 4 inhibitors)) OR TS=(lag 3 inhibitors)) OR TS=(tim 3 inhibitors)) OR TS=(Nivolumab)) OR TS=(Pembrolizumab)) OR TS=(Cemiplimab)) OR TS=(Tislelizumab)) OR TS=(Dostarlimab)) OR TS=(Toripalimab)) OR TS=(Retifanlimab)) OR TS=(Atezolizumab)) OR TS=(Avelumab)) OR TS=(Durvalumab)) OR TS=(Ipilimumab)) OR TS=(Tremelimumab)) OR TS=(Camrelizumab)) OR TS=(Sintilimab)) OR TS=(Zimberelimab)) OR TS=(Spartalizumab) | 525521 |
| #3 | #1 And #2 | 2625 |

| ***Cochrane*** | ***Search Strategy:*** |  |
| --- | --- | --- |
| #1 | Rectal Neoplasms OR Neoplasm, Rectal OR Rectal Neoplasm OR Neoplasms, Rectal OR Rectum Neoplasms OR Neoplasm, Rectum OR Rectum Neoplasm OR Rectal Tumors OR Rectal Tumor OR Tumor, Rectal OR Cancer of Rectum OR Rectum Cancers OR Cancer of the Rectum OR Rectal Cancer OR Cancer, Rectal OR Rectal Cancers OR Rectum Cancer OR Cancer, Rectum:ti,ab,kw | 10151 |
| #2 | (Immunotherapy OR immunotherapies OR immunotherapeutic OR immunotherapeutic agents OR checkpoint inhibitor OR checkpoint inhibitors OR immune checkpoint inhibitors OR checkpoint blockade OR checkpoint blockades OR programmed cell death protein 1 OR programmed cell death 1 OR programmed death 1 OR programmed death protein 1 OR programmed cell death receptor 1 inhibitor OR programmed cell death receptor 1 inhibitors OR anti pd 1 antibody OR anti pd 1 antibodies OR programmed cell death 1 ligand 1 OR programmed death 1 ligand 1 OR programmed death ligand 1 OR PD-1 OR PD1 OR PD-L1 OR PDL1 OR anti pd l1 antibody OR anti pd l1 antibodies OR anti pd l1 inhibitor OR anti pd l1 inhibitors OR pd 1 inhibitors OR pd l1 inhibitors OR ctla 4 inhibitors OR lag 3 inhibitors OR tim 3 inhibitors OR Nivolumab OR Pembrolizumab OR Cemiplimab OR Tislelizumab OR Dostarlimab OR Toripalimab OR Retifanlimab OR Atezolizumab OR Avelumab OR Durvalumab OR Ipilimumab OR Tremelimumab OR Camrelizumab OR Sintilimab OR Zimberelimab OR Spartalizumab):ti,ab,kw | 29415 |
| #3 | #1 And #2 | 263 |

| ***Embase*** | ***Search Strategy:*** |  |
| --- | --- | --- |
| #1 | ('Rectal Neoplasms' OR 'Neoplasm, Rectal' OR 'Rectal Neoplasm' OR 'Neoplasms, Rectal' OR 'Rectum Neoplasms' OR 'Neoplasm, Rectum' OR 'Rectum Neoplasm' OR 'Rectal Tumors' OR 'Rectal Tumor' OR 'Tumor, Rectal' OR 'Cancer of Rectum' OR 'Rectum Cancers' OR 'Cancer of the Rectum' OR 'Rectal Cancer' OR 'Cancer, Rectal' OR 'Rectal Cancers' OR 'Rectum Cancer' OR 'Cancer, Rectum') | 99246 |
| #2 | ('Immunotherapy' OR 'immunotherapies' OR 'immunotherapeutic' OR 'immunotherapeutic agents' OR 'checkpoint inhibitor' OR 'checkpoint inhibitors' OR 'immune checkpoint inhibitors' OR 'checkpoint blockade' OR 'checkpoint blockades' OR 'programmed cell death protein 1' OR 'programmed cell death 1' OR 'programmed death 1' OR 'programmed death protein 1' OR 'programmed cell death receptor 1 inhibitor' OR 'programmed cell death receptor 1 inhibitors' OR 'programmed cell death 1 receptor inhibitor' OR 'anti pd 1 antibody' OR 'anti pd 1 antibodies' OR 'programmed cell death 1 ligand 1' OR 'programmed death 1 ligand 1' OR 'programmed death ligand 1' OR 'PD-1' OR 'PD1' OR 'PD-L1' OR 'PDL1' OR 'anti pd l1 antibody' OR 'anti pd l1 antibodies' OR 'anti pd l1 inhibitor' OR 'anti pd l1 inhibitors' OR 'pd 1 inhibitors' OR 'pd l1 inhibitors' OR 'ctla 4 inhibitors' OR 'lag 3 inhibitors' OR 'tim 3 inhibitors' OR 'Nivolumab' OR 'Pembrolizumab' OR 'Cemiplimab' OR 'Tislelizumab' OR 'Dostarlimab' OR 'Toripalimab' OR 'Retifanlimab' OR 'Atezolizumab' OR 'Avelumab' OR 'Durvalumab' OR 'Ipilimumab' OR 'Tremelimumab' OR 'Camrelizumab' OR 'Sintilimab' OR 'Zimberelimab' OR 'Spartalizumab') | 521836 |
| #3 | #1 And #2 | 2573 |
