# Supplementary material for: Neoadjuvant chemoradiotherapy with or without PD-1 inhibitors in MMR−proficient non−metastatic rectal cancer: a meta-analysis of randomized controlled trials
Source: Front Immunol. 2026 Mar 3;17:1792283. doi: 10.3389/fimmu.2026.1792283 (PMC12992012; doi:10.3389/fimmu.2026.1792283)
Supplement: Supplementary file 4 [file Table2.docx]

Supplement Table 2. Sensitivity analysis of pCR

| Outcome | Method | RR (95% CI) | P-value | I² (%) |
| --- | --- | --- | --- | --- |
| pCR | DerSimonian–Laird | 0.58 (0.29–0.88) | 0.000 | 0.4 |
| pCR | Hartung–Knapp + REML | 0.58 (0.21–0.96) | 0.010 | 0.4 |
